# Single-Cell RNA-Seq of Bone Marrow-Derived Mesenchymal Stem Cells Reveals Unique Profiles of Lineage Priming

**DOI:** 10.1371/journal.pone.0136199

**Published:** 2015-09-09

**Authors:** Brian T. Freeman, Jangwook P. Jung, Brenda M. Ogle

**Affiliations:** 1 Department of Biomedical Engineering, University of Minnesota, Minneapolis, Minnesota, 55455, United States of America; 2 Stem Cell Institute, University of Minnesota, Minneapolis, Minnesota, 55455, United States of America; 3 Masonic Cancer Center, University of Minnesota, Minneapolis, Minnesota, 55455, United States of America; 4 Lillehei Heart Institute, University of Minnesota, Minneapolis, Minnesota, 55455, United States of America; University of Sao Paulo—USP, BRAZIL

## Abstract

The plasticity and immunomodulatory capacity of mesenchymal stem cells (MSCs) have spurred clinical use in recent years. However, clinical outcomes vary and many ascribe inconsistency to the tissue source of MSCs. Yet unconsidered is the extent of heterogeneity of individual MSCs from a given tissue source with respect to differentiation potential and immune regulatory function. Here we use single-cell RNA-seq to assess the transcriptional diversity of murine mesenchymal stem cells derived from bone marrow. We found genes associated with MSC multipotency were expressed at a high level and with consistency between individual cells. However, genes associated with osteogenic, chondrogenic, adipogenic, neurogenic and vascular smooth muscle differentiation were expressed at widely varying levels between individual cells. Further, certain genes associated with immunomodulation were also inconsistent between individual cells. Differences could not be ascribed to cycles of proliferation, culture bias or other cellular process, which might alter transcript expression in a regular or cyclic pattern. These results support and extend the concept of lineage priming of MSCs and emphasize caution for *in vivo* or clinical use of MSCs, even when immunomodulation is the goal, since multiple mesodermal (and even perhaps ectodermal) outcomes are a possibility. Purification might enable shifting of the probability of a certain outcome, but is unlikely to remove multilineage potential altogether.

## Introduction

Mesenchymal/multipotent stem/stromal cells (MSCs) are utilized in stem cell therapy for treatment of a variety of diseases including myocardial infarction, cancer, lung fibrosis, spinal cord injury, bone and cartilage repair, and muscular dystrophy[1–4]. MSCs are clinically beneficial due in part to the ability to home to sites of injury[5, 6], differentiate to mesenchymal cell types, suppress immune responses[7] and modulate angiogenesis[8–10]. In addition, MSCs are easy to isolate and expand and can be derived from multiple different tissue sources including bone-marrow, fat, placenta, synovium, periosteum, and tooth[2].

The large variety of tissue sources and species from which MSCs can be isolated have spurred efforts to characterize and compare each MSC isolate. The approach has been to identify a protein marker, or series of markers unique to MSCs and then to validate multipotency via differentiation protocols. For example, human MSCs are typically isolated from bone-marrow by selecting for adherent cells then confirming expression of CD73^+^/CD90^+^/CD105^+^/CD34^-^/CD14^-^/CD19^-^/CD45^-^ via a variety of methods including flow cytometry or fluorescence microscopy[11]. Use of the entire panel is inconsistent, as are the subsets selected by individual investigators[12, 13]. A similar trend occurs with isolation and characterization of murine MSCs derived from bone marrow. In this case, more than thirty different surface markers have been used with varying subsets over the past 15 years[14]. It is challenging to determine whether subset selection indicates an assumption by investigators that each subset reflects the whole or that a given isolate does not in fact express certain markers. But we do know that inconsistent use of MSC biomarkers to isolate “pure” populations can lead to variable levels of differentiation potential and ability to self renew[15, 16].

More perplexing is the fact that *consistent* use of biomarkers, can also lead to variable *in vitro* and *in vivo* outcomes between research groups. For example, murine bone marrow-derived MSCs sorted via immunodepletion of CD11b and CD45 for treatment of acute lung injury showed increased survival across studies but associated mechanisms were varied and sometimes contradictory[17–20]. Gupta et al., showed elevation of IL-10 and no change in neutrophil infiltration relative to controls[18], while Xu et al., showed no change in IL-10 production, but a decrease in neutrophil infiltration[17]. In addition, retention of water in the lungs was significantly decreased only after 48 hours in the study by Gupta et al., while in Xu et al., water retention was only observed at 24 hours and lost by 48. Some of this variation has been attributed to age or disease of the organism at the time of MSC isolation[21]. More likely however, is the fact that a handful of proteins cannot adequately describe the varied members of MSCs between species, between tissues and even perhaps between cells of a given population. Advanced molecular approaches including microarray[22], qPCR[23] and RNAseq[12, 13, 24–29] have allowed extensive characterization of 100s to 10,000s of transcripts of MSC populations. Results of these studies suggest heterogeneity of MSC populations could be ascribed to populations containing a varied mixture of undifferentiated MSCs primed[12, 30] for multiple pathways. Lineage priming has been observed with hematopoietic stems cells using single cell RT-PCR[31] and microarray[32, 33]. Transcription factors from both erythroid and myeloid differentiation pathways were expressed at a variety of levels in hematopoietic stems cells, suggesting that these stem cells could differentiate effectively down either pathway. Delorme et al., proposed that a similar process might occur with MSCs, but the study was conducted on clonal populations using qPCR and therefore a limited number of transcripts[12].

Until recently, cell-by-cell, whole-transcriptome analysis of MSCs has not been possible. However, advances in microfluidics and small volume cDNA synthesis now allow single-cell RNA-seq of individual MSCs[34]. Using this approach we find that individual MSCs exhibit multilineage priming, but priming is not uniform and appears to favor one and sometimes two lineages even while maintaining multipotency. In addition, a limited, but measureable degree of heterogeneity is observed in expression of genes associated with immunomodulation in the absence of immune stimulation. These results point to an as yet unappreciated source of heterogeneity of MSCs from a single tissue source.

## Materials and Methods

### Cell culture

Mouse bone marrow mesenchymal stem cells (mMSCs) were purchased (3 month-old male C57BL mice, Georgia Reagents University, Augusta, GA, all procedures approved by the Institutional Care and Use Committee, Medical College of Georgia[35]) and expanded and cultured as previously described[36]. Briefly, MSCs were cultured on a 0.1% gelatin (Sigma Aldrich, St. Louis, MO) pretreated flask containing α-minimum essential medium (MEM) complete. Complete alpha-MEM consisted of α-MEM (Invitrogen, Carlsbad, CA), 10% fetal bovine serum (Hyclone, Logan, UT), 0.1 mM nonessential amino acids (Invitrogen), and 2 mM L-glutamine (Invitrogren). MSC cultures were allowed to grow to 60–70% confluence and were replated at a concentration of 1,500 cells/cm^2^. Experiments were performed using passages 6–11 for mMSCs. As a control, HL-1 cardiomyocytes (HL-1cm) (a gift of Dr. William Claycomb) were also expanded and cultured as previously described[37]. All cultures were maintained at 37°C in 5% CO_2_.

### Single-cell capture and RNA-seq

mMSCs were trypsinized and suspended in phosphate buffered saline (PBS). The mMSCs were centrifuged and resuspended in 5 μL of complete alpha-MEM medium for addition to the capture chip. mMSCs were captured on a large-sized (17–25 μm cell diameter) chip using the Fluidigm C1 system. Cells were loaded into the chip at approximately 2000 cells/L and stained for viability (DEAD cell viability assay; Molecular Probes, Life Technologies, Grand Island, NY) and imaged with phase-contrast and fluorescence microscopy to confirm cell number and viability at each capture point. mMSCs and the HL-1cm controls were captured in the same Fluidigm C1 device with the HL-1cm labled with a green cytoplasmic dye (1 μm CellTracker Green CMFDA, Molecular Probes, Eugene, OR). 16 single, live mMSCs and 5 single, live HL1cm were selected for analysis. Once cells were captured in the device, cDNAs were prepared from each cell on the chip using the SMARTer Ultra Low RNA kit for Fluidigm C1 System (Clontech, Mountain View, CA). RNA spike-in Mix (Ambion, Life Technologies) was added to the lyses reaction and processed with cellular mRNA. mRNA library was constructed using the Illumina Nextera XT preparation kit (Illumina, San Diego, CA) according to the manufacturer’s protocol and sequenced on the Illumina MiSeqv3 using paired end reads with a length of 75 bp to a depth of 18 to 22 million reads with the Multiplex on one MiSeq lane to create *.fastq files. A bulk population RNA control of both mMSCs and HL-1cm was run in parallel to the single-cell samples. RNA-seq data were submitted to NCBI Gene Expression Omnibus and can be accessed via the following link, http://www.ncbi.nlm.nih.gov/geo/query/acc.cgi?acc=GSE70930.

### Gene expression analysis

Gene expression analysis was performed with Galaxy software (Minnesota Supercomputing Institute (MSI), University of Minnesota, Minneapolis, MN). Reads were processed and aligned to the mouse reference genome (mm10_genes_2012_05_23.gtf and canonical_mm10.fa) using Tophat (version 2.0.12, open source software, http://ccb.jhu.edu/software/tophat/index.shtml)[38]. See **S1 Table** for information about the total number of reads and percent concordant mapped reads for each cell. The default options supplied with the software were used and the aligned read files produced by Tophat were processed using Cufflinks software (version 2.2.1, open source software, http://cole-trapnell-lab.github.io/cufflinks/), for further analysis, including assembling transcripts, estimating their abundance, and testing for the differential expression between single-cell RNA-seq samples[38]. Read counts were normalized to fragments per kilobase of exon per million mapped reads (FPKM) according to the gene length and total mapped reads. Genes with a log_2_ fold change greater than 1 from the control cells to the fusion products and had a *P* value of less than 0.05 were considered “differentially expressed” and further analyzed for gene ontology (**S1 Table**). Gene ontology and Kyoto Encyclopedia of Genes and Genomes (KEGG) pathway enrichment analyses were performed with DAVID informatics resources 6.7 of the National Institute of Allergy and Infectious Diseases (NIAID) and of the National Institutes of Health (NIH)(**S3 Table**)[39, 40].

### Gene cluster analysis

Average linkage hierarchical clustering of gene expression intensity was performed using the Pearson distance to measure distance between gene and single cells. SingulaR (Fluidigm, San Francisco, CA) was used to compute and create the hierarchal clustering and principle component analysis plots (**S2 Table**).

### Statistical analysis

Data were analyzed with Microsoft Excel (Microsoft, Redmond, WA, USA). RNA-seq data was analyzed with the Cuffdiff or SingulaR programs.

### Quantitative real-time polymerase chain reactions (qRT-PCR)

Complementary DNA (cDNA) was synthesized following the instructions from the Maxima First Strand cDNA Synthesis Kit (cat# K1642, Thermo Scientific). The cDNA was amplified from the cDNA from the single-cell reaction performed in the Illumina chip. Primers of qRT-PCR were purchased from Biorad (Hercules, CA) and the sequence of *Gapdh* primers (FOR: CATGGCCTTCCGTGTTCCTA, REV: CCTGCTTCACCACCTTCTTGAT) was obtained from [41]. Primer efficiencies were extracted from RealPlex^2^ software and verified with melting curves. The comparative C_t_ method[42] was employed to determine the relative changes in gene expression. For comparison to the single-cell RNA-seq results, each gene’s FPKM values were normalized to each cells *Gapdh* FPKM value, which was then normalized to the control cells.

## Results

### Bone marrow-derived mMSCs express genes associated with MSC multipotency

**Fig 1:**
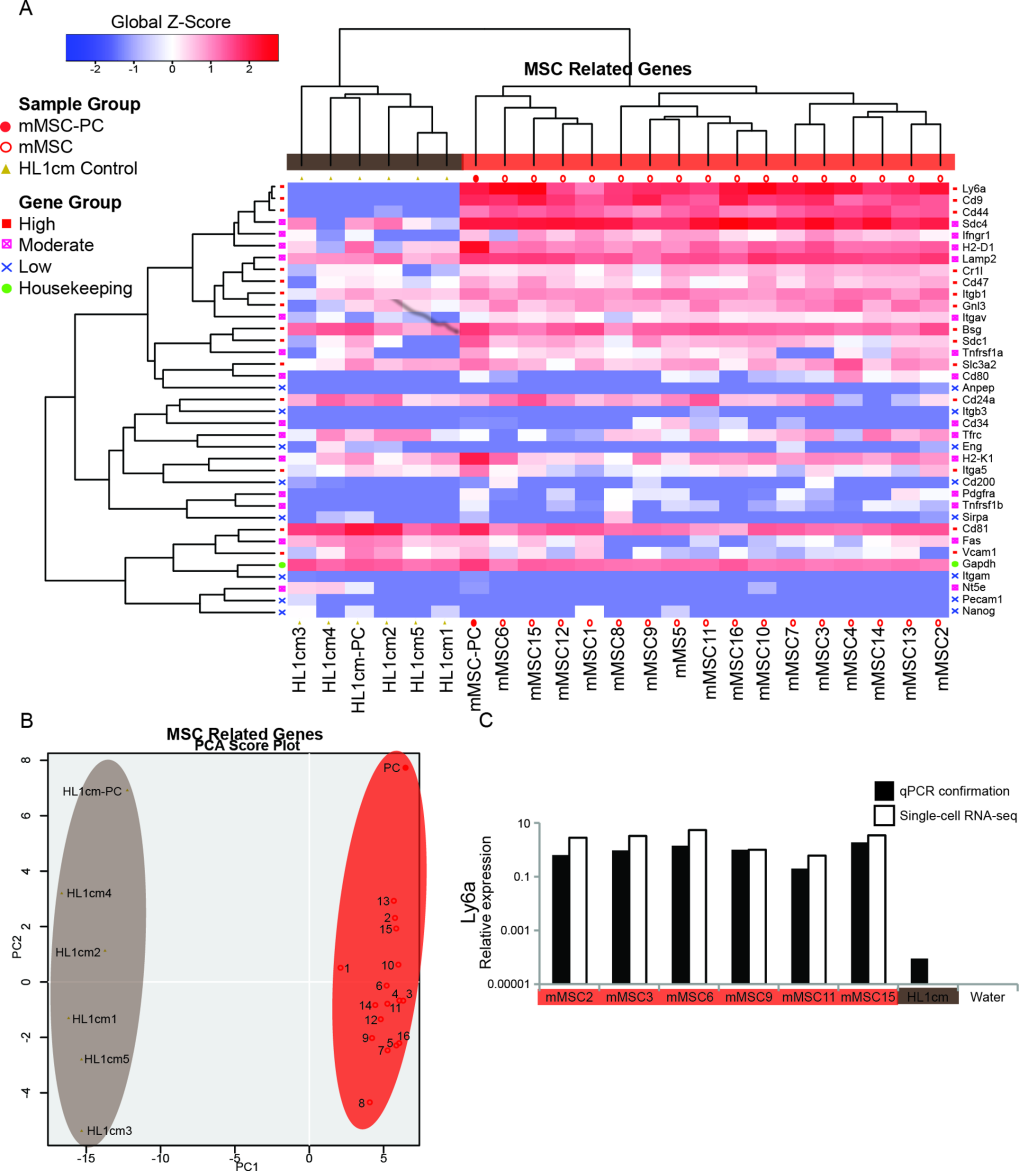
Single-cell mMSCs express genes associated with MSC multipotency **(A)** Hierarchal clustering (HC) of mMSCs for a set of genes related to MSC multipotency. Genes are organized either by genes expressed highly in mMSCs (red), genes expressed moderately/heterogeneously in mMSCs (pink), genes that are lowly expressed in mMSCs (blue) or housekeeping genes (green). Global Z-Score reflects the number of standard deviations away from the mean of expression in the reference. **(B)** Principal component analysis (PCA) analysis of single cell mMSCs (1–16) (red), negative control single cells (HL1cm1-HL1cm5) (brown), and population controls (PC and HL1cmPC). **(C)** Quantitative reverse transcriptase polymerase chain reaction (qPCR) confirmation of RNA-seq data.

To begin characterization of the transcriptome of bone marrow-derived MSCs, sixteen mouse mesenchymal stem cells (mMSC1-mMSC16) were individually sequenced using single-cell RNA-seq. Also, a population control (PC) containing thousands of cells (mMSC-PC) was sequenced in parallel with the single cells. Next, a gene set was assembled that included published markers indicative of MSC multipotency[14, 43, 44] (**S4 Table**). Hierarchal clustering (HC) and principal component analysis (PCA) analysis were then conducted using this gene set. HL-1 cardiomyocytes (HL1cm) were used as a negative control for this analysis and *Gapdh* was used to represent basal expression of a housekeeping gene. The mMSCs all clustered together far from the HL-1cm controls (**Fig 1A and 1B**). Of note, all mMSCs and mMSC-PC exhibited high expression of MSC stemness-associated markers previously shown to be upregulated in mouse MSCs (*Ly6a*, *Cd9*, *Cd44*, *Sdc4*, *Lamp2)* and low expression of MSC stemness-associated markers previously shown to be downregulated in mouse MSCs (*Anpep, Itgam, Eng, Nt5e, Pecam1, Nanog)[14]*. In addition, mMSCs exhibited inconsistent expression of markers reported to have variable expression in mMSCs (*Pdgfra, Cd80, Cd34, Tfrc)[14]*. The HL1cm control cells expressed MSC markers at a much lower level, but with similar levels of *Gapdh* expression, demonstrating the specificity of the gene set for mMSCs. RNA-seq data was confirmed with qPCR analysis of *Ly6a* showing similar relative expression between mMSCs and low expression in the HL1cm control (**Fig 1C**).

### Multilineage priming of individual bone marrow-derived mMSCs

**Fig 2:**
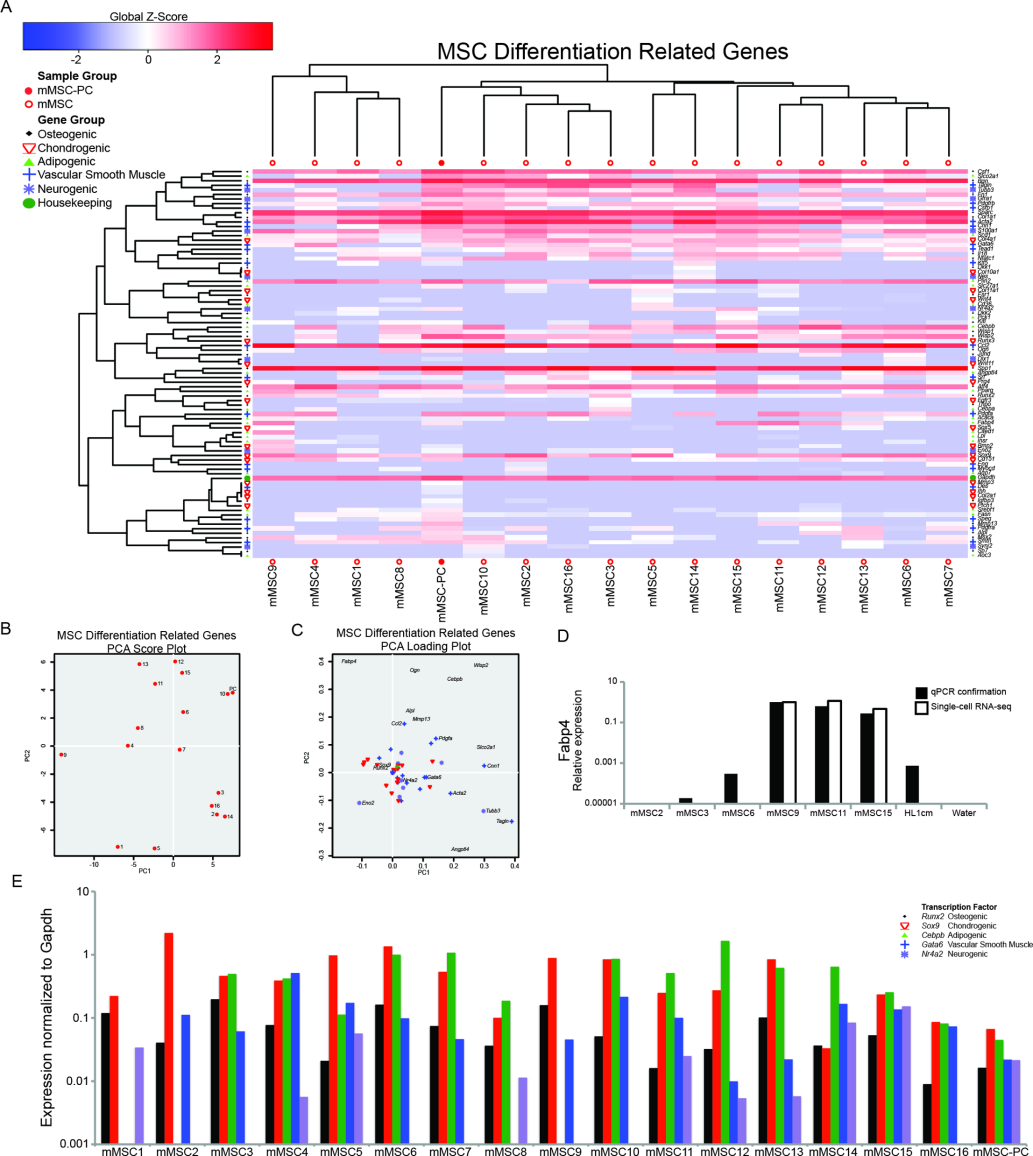
Individual mMSCs express genes from multiple differentiation pathways. **(A)** HC of single cell mMSCs and mMSC population control (mMSC-PC) for a set of genes related to osteogenesis, chondrogenesis, adipogenesis, vascular smooth muscle, neurogenesis, and housekeeping genes. **(B)** PCA analysis of single cell mMSCs (1–16) and population control (PC). **(C)** PCA loading plot showing individual differentiation genes represented in **(B)**. **(D)** qPCR confirmation of RNA-seq data. **(E)** A key transcription factor was selected for each differentiation lineage and expression was normalized to *Gapdh* for each sample.

To determine whether differentiation potential could account, at least in part, for varied *in vivo* outcomes, we generated gene sets corresponding to mesenchymal specification[12, 45–53]. The gene list included early stage (e.g. transcription factors) and late stage (e.g. functional proteins) markers for osteogenic, chondrogenic, adipogenic, vascular smooth muscle and neurogenic lineages. Using this set, HC and PCA analyses were conducted on single-cell mMSCs and the mMSC-PC (**Fig 2A–2C**). The mMSC-PC control correlated with the average of the single mMSCs supporting the accuracy of the single-cell data. In addition, the population control contained transcripts corresponding to early and late markers of multiple lineages at variable levels, a finding perhaps attributable to the mixing of multiple lineage-committed or lineage-primed populations. The profile of the mMSC-PC control was similar to that of each single-cell mMSC (**Fig 2A**). Individual mMSCs did not express markers of a single lineage, but instead showed expression of early stage markers of at least three differentiation pathways *simultaneously* (**Fig 2E** and **S1 Fig**). Since it was previously established that all mMSCs used in this study were still expressing high levels of multipotency-associated genes (**Fig 1A and 1B**), this was quite surprising. The separation of individual MSCs in the PCA plot was due to variable expression of genes from all five lineages. Cells at the top of the PCA score plot had higher levels of a few osteogenic (*Ogn* and *Wisp2*) and adipogenic (*Fabp4* and *Cebpb*) markers, cells on the right had higher levels of vascular smooth muscle markers (*Acta2*, *Cnn1* and *Tagln*), *Tubb* (neurogenic), *Slco2a1* and *Angptl4* (most often associated with adipogenesis) (**Fig 2C**). In an attempt to more easily visualize the varied linage propensities of individual mMSCs, one key transcription factor for each lineage (*Runx2*, *Sox9*, *Cebpb*, *Gata6* and *Nr4a2*) was selected and normalized to *Gapdh* expression for all sixteen cells and the population control (**Fig 2E**). The population control revealed a similar profile to that seen for the clonal populations of the Delorme study[12], but surprisingly each individual mMSCs exhibited much more varied profiles. Seven of the sixteen mMSCs expressed all five transcription factors, six mMSCs expressed four of the transcription factors and three mMSCs expressed three of the transcription factors. These data were confirmed with qPCR of a gene highly expressed in all mMSCs (*Ly6a*) (**Fig 1C)** and a gene with high variability of expression in individual mMSCs (*Fabp4*) (**Fig 2D**).

### Basal immunomodulatory capacity of individual bone marrow-derived mMSCs

**Fig 3:**
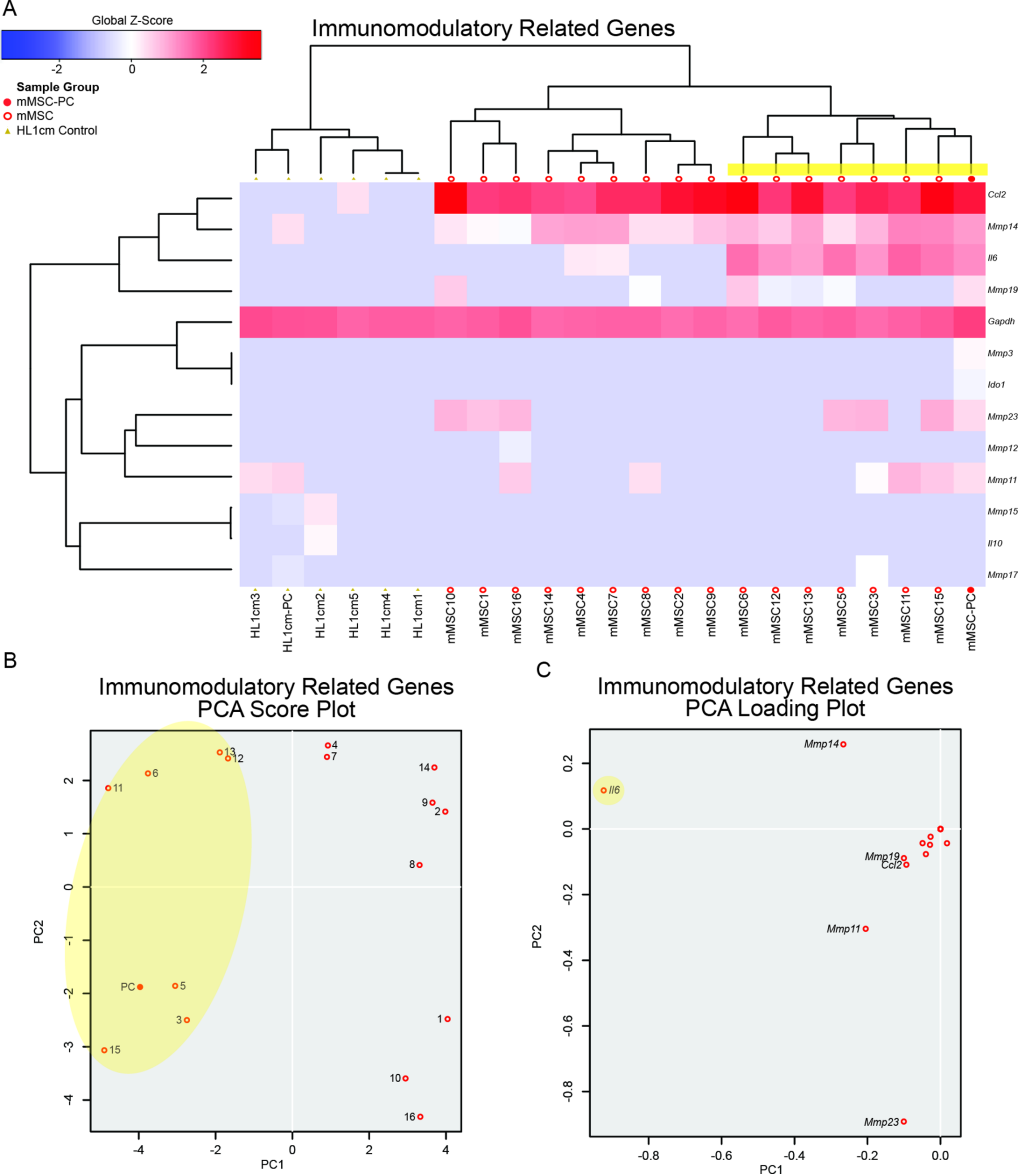
Single cell mMSCs exhibit minimal but measureable differences in expression of immunomodulatory genes. **(A)** HC of single cell mMSCs, negative control single cells (HL1cm1-5), and population controls (mMSC-PC and HL1cmPC) for a set of genes related to immunomodulatory function. **(B)** PCA analysis of single cell mMSCs (1–16) and population control (PC). **(C)** PCA loading plot showing individual differentiation genes represented in **(B)**. *Il6* expressing cells clustered together (yellow).

To determine whether basal immunomodulatory capacity could also account for varied MSC behavior *in vitro* and *in vivo*, we generated a gene set containing soluble, immunoregulatory factors known to be produced by MSCs *in vitro* and *in vivo*[54–56] (**Fig 3A, S4 Table**). Individual cells were largely the same with the exception of *Il6* expression. Indeed variability in the expression of this gene accounted almost solely for the population spread on the PCA score plot (**Fig 3B and 3C**). The significance of this difference was further probed via global analysis of differentially expressed genes.

### Transcriptome heterogeneity of mMSCs

**Fig 4:**
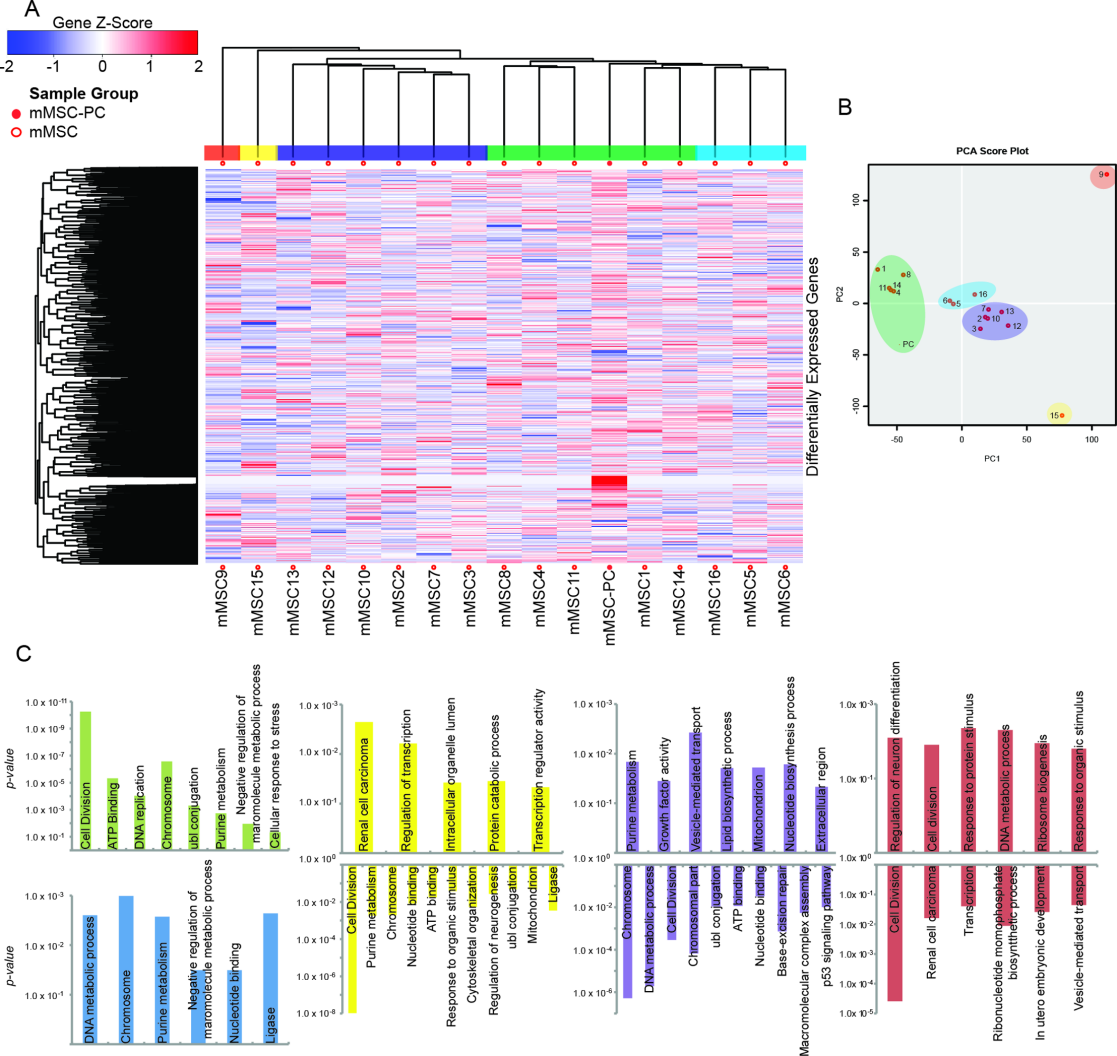
HC and PCA of mMSCs show transcriptome heterogeneity and gene ontology of subpopulations. **(A)** A global view of differential gene expression between single cell mMSCs and the population control (mMSC-PC). Gene Z-Score reflects the number of standard deviations away from the mean of expression in the reference. **(B)** PCA analysis of single cell mMSCs (1–16) and population control (PC). **(C)** Gene onotology of five subpopulations of single-cell mMSCs (green, blue, yellow, purple and red clusters). Ontology groups are plotted with the *P* value on the y-axis. Upregulated groups have bars extending in the positive y-direction and down regulated groups have bars extending in the negative y-direction.

To more comprehensively delineate differences in the transcriptome that might augment findings related to lineage priming and support/refute subtle differences in immunomodulatory function, we identified all differentially expressed genes between each mMSC as well as the population control. Differential expression was defined as a log_2_ fold change of greater than 1 and a *P* value below 0.05. Over 9,000 differentially expressed genes were identified and displayed using HC (**Fig 4A**) and PCA score plot (**Fig 4B**). Results indicate global heterogeneity of single mMSCs arguably expected of mesenchymal progenitors (**Fig 4A and 4B).** Despite the heterogeneity, five different clusters emerged and are designated with different color labels. At first glance, we suspected clusters might represent different types and levels of lineage commitment of mesenchymal progenitors. To test this possibility, gene ontology analysis was conducted on the differentially expressed genes to determine whether each cluster aligned with specific mesenchymal differentiated cell types (e.g., osteogenic, adipogenic, chondrogenic).

DAVID bioinformatics resource was utilized to define function-related gene groups associated with each cluster of MSCs. Differentially expressed genes were isolated for each cluster of MSCs and separated according to up- or down-regulation (**S3 Table**). The significance of each concentrated gene group was plotted on a negative log scale with upregulated gene groups extending upward and downregulated gene groups extending downward (**Fig 4C**). Only gene groups with a *P* value below 0.05 were included in the graph and surprisingly only one was related to differentiation. The first cluster (green) contained upregulation of genes involved in cell division, ATP binding, DNA replication, and chromosomes which suggests that this cluster of five cells (along with the population control) was in active proliferation at the time of cell capture. The ratio of cells (5 of 16, ~30%) matches our data and literature reports of the percentage of cells actively dividing in a healthy population of bone marrow-derived MSCs[57]. The blue cluster showed upregulation of genes associated with DNA metabolic process, chromosomes, purine metabolism and nucleotide binding. The blue cluster did not contain genes upregulated with cell division, but these three cells do appear to be preparing DNA for future division. The cell of the yellow cluster (mMSC15) upregulated genes associated with regulation of transcription and protein catabolic process, while downregulating genes associated with cell division and regulation of neurogensis. The purple cluster also downregulated genes linked to cell division, but upregulated genes involved with growth factor activity, vesicle-mediated transport, lipid biosynthetic process and the extracellular region. This cluster could perhaps represent active paracrine signaling corresponding to the immunomodulatory capacity of MSCs. If active paracrine signaling is defined as augmented *Il6* production (**Fig 3**, mMSC 3, 6, 5, 11, 12, 13, 15), then there is an insignificant relationship between active paracrine signaling and association with the purple cluster (Chi-square statistic 0.15, *P* = 0.70). Indeed, a relationship with similar strength exists between proliferation and downregulation of *Il6* (Chi-square statistic 2.14, *P* = 0.14). Instead, the purple cluster could represent a subpopulation of mMSCs with advanced commitment to adipogenesis, since this cluster also shows an upregulation of the lipid biosynthetic process. However, cross reference of cells of this group (mMSC2, 3, 7, 10, 12 and 13) to the differentiation analysis (**Fig 2A–2B**), shows each falls in different regions of the PCA plot suggesting they each have unique lineage priming, not significantly tending toward adipogenesis. The cell of the red cluster (mMSC 9) showed an unexpected upregulation of genes involved with the regulation of neuronal differentiation and ribosome biogenesis, suggesting priming for neurogenesis which has been reported in isolated instances for MSCs[45–47]. In sum, mMSC clusters primarily correspond to proliferating cells (green and blue clusters) and quiescent cells (yellow, purple and red clusters). Importantly proliferation status does not correlate with the level or number of lineage commitment or immune regulatory genes expressed per cell.

## Discussion

In this study the transcriptomes of individual, bone marrow-derived mMSCs were analyzed via RNA-seq. Using this approach, a new perspective on the heterogeneity of MSCs emerges. First, MSCs were found to express varied levels of early markers of multiple mesenchymal lineages extending the definition of lineage priming of this unique cell type. Importantly, differences in level or number of lineage commitment genes expressed did not correlate with proliferation or other cellular process, which might alter transcript expression in a regular or cyclic pattern. Second, basal expression of genes associated with immunomodulation were quite uniform aside from *Il6* in gene set analyses and categories related to immunomodulation did not emerge from ontology analyses suggesting cell-to-cell variation for immunomodulation is present but less pronounced than lineage commitment.

Since there was no clear functional clustering of the mMSCs based on gene ontology (aside from proliferating/nonproliferating), we also scanned for gene markers that could be used to represent the clustered populations (**Fig 2**) and perhaps correspond to lineage priming. A previous report on mouse MSCs subpopulations found that *Cd200* expression level indicated osteogenic potential and *Pdgfra* expression indicated adipogenic potential[14]. However, mMSC3, mMSC6, and mMSC9 were the only mMSCs with detectable *Cd200* expression and indeed these three mMSCs did not cluster in either the global analyses (**Fig 4**) or gene list analyses with markers of differentiation (**Fig 2**). *Pdgfra* expression also failed to aid clustering of the eleven mMSCs that had detectable expression (mMSC2, 3, 5, 6, 7, 8, 9, 10, 12, 13, 14). The discrepancy between our work and previous reports suggests an environmental trigger was necessary to promote particular cell fates and therefore that marker expression alone (at least in these two cases) is not enough to predict single cell propensities. We also note, based on the results presented here, that inter-lineage plasticity is likely very high. And that, even if an MSC “commits” to a particular lineage, conversion to another might not entail the set up of whole molecular programs, but instead upregulation of a few components[58].

Of note, one cell upregulated expression of neurogenic transcript for *Eno2*. This is not the first report of an MSC expressing transcripts considered specific to ectodermal lineage. In multiple reports Foudah *et al*. demonstrate that neuronal makers *Tubb* and *NeuN* are spontaneously expressed by a high percentage of undifferentiated MSCs from multiple tissues from both human and rat[45–47]. Thus, while the relative fraction of MSCs capable of expressing early transcripts of neurogenesis and of giving rise to bona fide neurons is not known, single cell analysis suggests plasticity to this lineage may be possible.

In sum, this study confirms and augments the concept of multi-lineage priming of MSCs by showing that MSCs typically express early (and sometimes even late) markers of more than one mesenchymal lineage *simultaneously*. Moreover, expression levels are quite distinct between cells, suggesting MSCs, despite being primed to multiple fates, might prefer one fate over the other. In other words, even if an MSC is environmentally triggered for one lineage, it might easily be switched if even a weak signal comes along for the preferred lineage. Immunomodulation of MSCs on the other hand, appears to be relatively consistent between cells of a given population. Even so, these results emphasize caution for *in vivo* or clinical use of MSCs, even when immunomodulation is the goal, since multiple mesenchymal (and even perhaps ectodermal) outcomes are a possibility. Purification might enable shifting of the probability of a certain outcome, but can never remove multilineage potential altogether.

## Supporting Information

S1 Fig Expression of each lineage gene normalized to *Gapdh*. **(A).** Osteogenic gene profile. **(B)** Chondrogenic gene profile. **(C)** Adipogenic gene profile. **(D)** Vascular smooth muscle gene profile. **E**. Neurogenic gene profile.
(TIF)

S1 Table Alignment statistics for single-cell with sequenced transcriptome, FPKM values and differentially expressed genes of mMSC clusters, Related to Fig 4. This first tab contains all of the alignment statistics for the captured mMSCs and HL1cm controls relative to the mouse genome. The number of aligned read pairs and the percent of concordant alignment (or reads with proper alignment) are listed for each single cell. The alignment data were generated from Tophat alignment results. The second tab is the single-cell RNA-seq expression data (FPKM values) for the mMSCs (16 cells and population control) and HL-1-cardiomyocytes controls (5 cells and population control) in an excel file as discussed in **Figs 1–4**. The remainder of the file contains all of the differentially expressed genes detected with the Cufflinks package analysis between the five mMSC clusters. The file contains the gene (columns A-C), locus (column D), samples compared (columns E and F), the FPKM values (H and I), the log_2_ fold change (column J), *P* value (column L) and whether or not the differential expression is significant (column N). Genes are listed alphabetically and each mMSC cluster is separated onto different tabs.
(XLSX)

S2 Table SingulaR script used to analyze single-cell RNA-seq data. This file contains the SingulaR script used with the single-cell RNA-seq data to create the hierarchal clustering and principal component analysis.
(XLSX)

S3 Table Gene Ontology and KEGG pathway enrichment analysis, Related to Fig 4. This file contains all of the differentially expressed genes between the mMSC clusters, but organized into lists used for gene ontology on the first tab. The first tab is organized into upregulated and downregulated genes for each cluster of mMSCs. The remaining tabs contain the gene ontology and KEGG pathway enrichment analysis that was performed using DAVID informatics Resources 6.7 for upregulated or downregulated differentially expressed gene lists for mMSCs clusters as identified in the Cufflinks differential analysis.
(XLSX)

S4 Table Genes Lists, Related to Fig 4. This file contains the genes associated with each gene list (MSC stemness[14, 43, 44], MSC Differentiation[12, 45–53], and immunomodulatory[54–56]. Included in this file are the gene symbol, full gene name, functional group and source for inclusion.
(XLSX)
